# The Role of the Immune Response in Age-Related Macular Degeneration

**DOI:** 10.1155/2013/348092

**Published:** 2013-05-23

**Authors:** Scott M. Whitcup, Akrit Sodhi, John P. Atkinson, V. Michael Holers, Debasish Sinha, Bärbel Rohrer, Andrew D. Dick

**Affiliations:** ^1^Allergan, Inc., 2525 Dupont Drive, Irvine, CA 92612, USA; ^2^The Wilmer Eye Institute, The Johns Hopkins University School of Medicine, Baltimore, MD 21287, USA; ^3^Division of Rheumatology, Washington University School of Medicine, St. Louis, MO 63110, USA; ^4^Division of Rheumatology, University of Colorado School of Medicine, Aurora, CO 80045, USA; ^5^Department of Ophthalmology, Medical University of South Carolina, Charleston, SC 29425, USA; ^6^School of Clinical Sciences and School of Cellular and Molecular Medicine, University of Bristol and Bristol Eye Hospital and NIHR, Bristol BS1 2LX, UK; ^7^Biomedical Research Centre at Moorfields Eye Hospital, NHS Foundation Trust, and UCL Institute of Ophthalmology, London EC1V 2PD, UK

## Abstract

Age-related macular degeneration (AMD) is the leading cause of blindness in developed countries; with the aging population, the negative health impacts and costs of the disease will increase dramatically over the next decade. Although the exact cause of AMD is unknown, genetic studies have implicated the complement system as well as other immune responses in disease pathogenesis and severity. Furthermore, histologic studies have shown the presence of macrophages, lymphocytes, and mast cells, as well as fibroblasts, in both atrophic lesions and with retinal neovascularization. This review summarizes discussions from the fifth annual conference of the Arnold and Mabel Beckman Initiative for Macular Research by the Inflammation and Immune Response Task Force. These deliberations focused on the role of inflammatory immune responses, including complement, inflammasomes, adaptive immune responses, and para-inflammation, unanswered questions and studies to address these questions, and potential immune-related therapeutic targets for AMD.

## 1. Introduction

Age-related macular degeneration (AMD) is the leading cause of central vision loss in developed countries. The most recent data suggest that more than 3 million people in the United States will be affected by the disease by 2020 [1]. The disease affects the choriocapillaris, Bruch's membrane and the retinal pigment epithelium, with dysfunction and death of overlying photoreceptors. In addition to age, risk factors for the disease include both environmental and epidemiologic factors. Specific disease associations include smoking, light exposure, obesity, and race [2]. Recent genetic studies have implicated roles for the immune system, particularly abnormalities in the complement system, in disease pathogenesis, and severity. Although patients with AMD do not have signs of overt ocular inflammation, histologic studies have shown the presence of macrophages, lymphocytes, and mast cells, as well as fibroblasts, associated with both atrophic lesions and with neovascularization of the retina [3].

 Importantly, the retina is a highly metabolically active tissue, with requirements to mediate photoreceptor turnover. As the retina ages, it may be less able to handle these metabolic requirements. Immunologically active deposits called drusen that contain lipids, complement, and other potentially immune activating substances may act as additional triggers for immune responses in the eye. Other inflammatory initiators include oxidative stress and secondary mediators of inflammation such as cytokines. On the other hand, the retina performs well until late in life despite constant stress, suggesting that at least some of the inflammatory responses observed may be beneficial. Equally intriguing, although perhaps less well understood, is a renewed appreciation for the role of the adaptive immune response in the pathogenesis of AMD. Collectively, as a result of previous studies showing inflammatory cells associated with AMD and newer genetic studies implicating the innate immune system in developing the disease, there is heightened interest in studying the role of the immune response in AMD and in determining whether modulating the immune response could help treat the disease.

 The extent to which innate and adaptive immune responses play roles in the pathogenesis of AMD, and the ability to target these pathways to effectively treat the disease, remains debatable. This may in part be due to the complexity of the immune response, the number of different inflammatory cell types and cytokines involved, and the kinetics of the inflammatory response. Further, it is as yet difficult to know whether immune responses are driven and controlled locally in the retina, or operate systemically, further complicating interpretations and the development of useful therapeutic approaches.

 One key question, however, is whether this immune activation is always pathologic in AMD, or whether it can actually help preserve function and moderate damage at certain stages of the disease. The data support the idea that activated states confer protection. Resident CD200R myeloid cells in the retina are under tonic control by cognate interaction with CD200 [4, 5]. The tissue consequence of microglial activation is context dependent [6, 7]. For example, in photoreceptor neurodegenerative models, microglia do not contribute to the progression of disease despite being activated [8]. In more inflammatory scenarios, a recognized consequence of activated response is contributing toward immune regulation in an attempt to contain further retinal damage [9]. A chronic inflammatory state has also been identified in a number of nonocular diseases, including type 2 diabetes and cardiovascular disease. Could a low-grade immune response be helpful in some circumstances? The intriguing concept has been distilled and developed to infer that tissue stress or malfunction can induce an advantageous response, and has been referred to as para-inflammation [10]. Medzhitov hypothesized that a well-controlled “para-inflammatory” response could be beneficial by either protecting against infection or preserving function in diseased tissues. The experimental evidence and now the concept of para-inflammation have been further articulated and illuminated experimentally by Xu et al., who discuss the potential role of para-inflammation in the aging retina elsewhere [11]. Briefly, and discussed in more detail below, immune activation and recruitment of macrophages may be required to help process photoreceptor and RPE byproducts, thus controlling overt inflammation, tissue dysfunction, and cell death.

 In January 2013, the fifth annual conference of the Arnold and Mabel Beckman Initiative for Macular Research was particularly focused on a common form of AMD, namely, atrophic macular degeneration. Meeting participants were divided into task groups devoted to discussing and brainstorming particular aspects of AMD, including one responsible for considering the role of inflammation and immune responses. This review arose in part from the discussions of that task group. Here, therefore, the role of immune responses in regulating or promoting tissue damage, including complement, inflammasomes, and para-inflammation, will be discussed, followed by a summary of the group's thinking on potential research approaches and therapeutic targets.

## 2. The Complement System and AMD

 The complement system is the most widely accepted pathogenic pathway of the immune system implicated in AMD. The genetic evidence from genome wide association studies (GWAS) and rare variant analyses indicate an overactive alternative pathway (AP). Multiple outstanding reports have detailed and reviewed this evidence at the genetic, RNA and protein levels [13, 14, 16–25]. Therefore, these data will primarily be summarized here—the underlying thesis being that excessive engagement of the alternative pathway is a key component in AMD pathogenesis.

 In 2005, four GWAS demonstrated that approximately 50% of the inheritance in AMD could be accounted for by a single nucleotide polymorphism (SNP) in an exon encoding the regulator complement factor H (CFH) [26–29]. Moreover, this SNP in CFH at amino acid position 402—a tyrosine (Y) (major allele) or a histidine (H) (minor allele)—has a functional consequence. At sites of tissue injury, the risk variant 402H does not dampen the alternative pathway (AP) of complement activation as efficiently as 402Y [30–34]. While the complement system had been previously implicated in AMD [35–38], it was the GWAS-derived genetic data that cemented the relationship [26–29]. 

**Figure 1:**
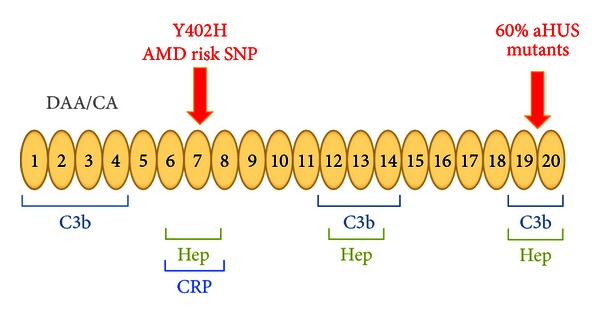
Schematic diagram of complement factor H (CFH). The protein consists entirely of 20 repeating homologous units (complement control repeats or CCPs), each ~60 amino acids in length (like beads on a string). The N-terminal portion houses the regulatory domains (repeats 1–4). The surface-binding recognition motifs are located in repeats 6–8, 12–14 and 19 and 20. They are also known as anionic- or heparin-binding sites. Both Y402H in repeat 7, and a rare variant in repeat 20 [12], are associated with AMD; these regions mediate the binding of factor H to cellular debris such as drusen or damaged retinal cells/tissues. Atypical hemolytic uremic syndrome (aHUS) has been compared to AMD because multiple variants leading to haploinsufficiency of factor H allow for excessive complement activation in this thrombomicroangiopathy [13–15]. Specifically, about 60% of the mutations in aHUS occur in repeats 19 and 20, which decrease factor H's ability to bind to damaged endothelium. DAA, decay accelerating activity; CA, cofactor activity; Hep, heparin binding; CRP, C-reactive protein. Modified from Richards et al. [13].

 In Caucasian populations of European ancestry, the risk allele (402H) has a gene frequency of 0.3 to 0.4, and the more common allele (Y402) 0.6 to 0.7. The 402H allele is likely replacing the major one because in early life it provides a survival advantage against streptococcal infections [13, 39, 40]. Multiple bacteria and several groups of viruses impair the complement system by hijacking the host's regulators (reviewed in [40]); for example, microbes bind CFH to their surface to inhibit complement activation. The CFH binding protein of group A beta hemolytic streptococcus has a lower affinity for 402H than for Y402. Consequently, the host's complement system has greater activity against the pathogen if the host expresses 402H, thereby reducing the microbes' ability to counteract the AP. CFH adheres to damaged eukaryotic cells and tissue debris via the same anionic (heparin) binding sites that microorganisms employ to attach it to their surface. Two and possibly as many as four such cellular and tissue binding sites are positioned along the linear CFH protein (Figure 1). An unintended consequence later in life of carrying 402H is that it does not bind as well as Y402 to debris in the retina. Differential binding of 402H versus Y402 to multiple constituents of a damaged retina [30–34, 41–44] has been demonstrated for DNA, RNA, lipids, C-reactive protein (CRP), necrotic and apoptotic cells, heparin and other glycosaminoglycans, lipofuscin, bisretinoids, photooxidation byproducts, and amyloid beta. The common finding is that the 402H protein binds with a lower affinity than Y402. Therefore, in the retina of an individual carrying this risk variant, there is a greater degree of AP activation as retinal debris accumulates in AMD patients.

 Thus, the complement hypothesis for the etiopathogenesis of AMD centers on the concept of an “overreaction” to injury and debris in the retina by individuals carrying a “complement hyperinflammatory phenotype” [13, 14, 45]. The AP becomes engaged on a target site if there is a relative lack of inhibitors. To regulate the AP that is continuously turning over, CFH must first transfer from plasma to the foreign material or altered self. To maintain homeostasis and to prevent excessive AP activation, it binds to the target using both C3b and anionic material as ligands for its multiple binding sites (Figure 1). It then serves as a cofactor for the serine protease (Factor I) to cleave C3b. This results in a C3 fragment that does not support amplification via the AP's feedback loop. A host carrying the 402H allele or other risk factors may deposit undesirable quantities of C3b and release C3a in the retina, as well as the downstream effectors C5a and C5b-C9. This scenario for AMD pathogenesis does not preclude triggering of the classical or lectin complement pathways by autoantibodies or lectins, which could then be followed by excessive amplification through the AP. Also, environmental (e.g., smoking) and endogenous (e.g., increased body mass) factors further tip the balance in favor of more inflammation [46–52].

 Multiple other CFH variants, both common and rare, influence risk of developing AMD [18, 20, 21, 53–55]. For example, the CFH 62I variant is protective, as is a 84 kb deletion of two CFH-related genes, FHR-1 and FHR-3. The simplest and most likely interpretation of these data is that these genetic changes enhance regulation of the AP by CFH. In contrast, a rare and defective CFH variant confers substantial risk (with high penetrance) for AMD [12]. This recent discovery of a rare variant in CFH with a large effect is probably just the beginning in terms of identification by targeted deep sequencing of highly penetrant mutations in regulators and components of the AP in AMD. Further, haploinsufficiency of C9 conferred a nearly 5-fold reduction in neovascular AMD in the Japanese population, where a nonsense mutation in the C9 gene is frequently found [56]. The interpretation here is that membrane attack complex is less active and thus is protective against retinal damage.

 In addition to risk and protective variants of CFH and CFH-related genes, polymorphisms in AP components C3 [57, 58] and factor B [30, 58] are also associated with AMD. A consistent observation is that the protective variants result in less AP activity, whereas risk variants result in more AP activity. Genetic variants in Factor I, the protease employed by CFH to inactivate C3b, have also been associated with AMD by GWAS [59]. Taken together, these findings provide powerful evidence implicating overactivation of the AP predisposing to AMD; thus, common and rare variants in multiple members of a proinflammatory pathway of innate immunity—the AP—are associated with the same disease. Those that decrease function of the pathway are protective, and those that increase function create risk. Moreover, the variants have both independent and additive effects on the risk of developing AMD [25, 47, 48, 52, 58].

 Other inhibitors of the AP include membrane cofactor protein (MCP; CD46), decay accelerating factor (DAF; CD55), and complement receptor one (CR1; CD35, the C3b/C4b or immune adherence receptor). MCP and DAF are widely expressed, whereas CR1 has a more limited distribution. DAF and MCP are expressed on the cell surface, where they protect healthy cells from complement attack. MCP is expressed at a high level by RPE cells (particularly at the basal surface) and endothelial cells [24]. A decrease in MCP expression at this RPE location was observed in early AMD. CR1 also has potent regulatory activity for AP C3 and C5 convertases. A surprising recent observation is CR1 expression on the apical surface of RPE cells [24]. These observations concerning the expression and function of DAF, MCP, and CR1 in the retina require further investigation. Although GWAS have not implicated DAF, MCP, or CR1 in susceptibility to AMD, results of targeted next generation deep sequencing of these genes have not been reported.

 A role for complement is further evident specifically in “wet” AMD. This severe condition is associated with choroidal neovascularization (CNV) [53], a process characterized by newly formed and leaky vessels invading the sub-retinal space. CNV is associated with fluid accumulation and retinal detachment with loss of the underlying photoreceptors. One animal model of wet AMD is the laser-induced CNV model in rodents. The model is initiated by argon laser photocoagulation, which ruptures Bruch's membrane and triggers complement activation [60]. In mice, the key role of the complement system in the development of CNV is well established. Using knockout and specific inhibitor approaches, it appears that the alternative pathway of complement is the key driver of CNV, in that the removing of the classical or lectin pathway has no protective effect [61, 62]. However, the alternative pathway alone is not sufficient to drive CNV, confirming its importance in the amplification loop [61]. With regard to effector functions, the anaphylatoxins C3a and C5a [60] are important in developing injury. In addition, the membrane attack complex (MAC) contributes to the development of CNV, as *CD59* ^−/−^ mice lacking the MAC regulator CD59 develop CNV at a higher level than control mice [63], and treatment with recombinant soluble CD59a-IgG2a fusion-protein [64] or gene therapy expressing soluble CD59 [65] both reduce CNV. The CNV model has also been successfully treated with the targeted murine CR2-factor H (muCR2-fH) protein, which consists of a domain which directs the regulatory domain of CFH to sites of complement activation [66], as demonstrated by systemic administration and evaluation of local CNV development [67]. Importantly, in each model evaluated, complement activation amplifies the generation of vascular endothelial growth factor (VEGF), which is strongly implicated in fueling the development of CNV and AMD [68].

## 3. Inflammasome Activation in AMD

 The maintenance of the delicate balance between self and nonself regulates cellular homeostasis. However, during the aging process this system may be more vulnerable to a variety of noxious challenges that may activate host defense systems. The inflammasome is responsible for activation of many inflammatory processes. The inflammasome is a multiprotein complex, comprising of a sensor protein, the adaptor protein ASC (apoptosis-associated speck-like domain containing a caspase recruitment domain), and the inflammatory protease caspase-1. The assembly of the inflammasome signaling platform occurs due to conformational changes in the sensor protein, which in turn recruits caspase-1 to the complex and subsequently promotes the activation of caspase-1. Once activated, caspase-1 cleaves the inactive precursors of two proinflammatory cytokines, interleukin 1*β* (IL-1*β*) and IL-18, thereby generating mature forms which are then secreted from cells [69]. The inflammasome forming sensors are different receptor molecules, such as nucleotide-binding domain and leucine-rich repeat containing family pyrin (NLRP), which belong to the Nod-like receptor family of proteins. These include NLRP1, NLRP3, and NLRC4; or Absent In Melanoma (AIM 2), a receptor of the HIN (IFN-inducible nuclear proteins) family of proteins [70]. A growing body of evidence suggests that the NLRP3 inflammasome is clearly involved in host defense and autoinflammatory conditions, and is an integrator of cell damage and stress signals [71].

 Activation of IL-1*β* by an inflammasome is required to efficiently control viral, bacterial, and fungal pathogen infections. However, excess IL-1*β* activity contributes to a variety of diseases [72]. The NLRP3 inflammasome has been shown to play a central role in the pathogenesis of autoinflammatory disorders; its activity has also been implicated in diseases such as Alzheimer's disease, cancer, type II diabetes, and most recently AMD [71, 73, 74]. The classic pathology of AMD is multiple small or intermediate drusen in the macular area. In a recent study, drusen isolated from donor AMD eyes were shown to activate NLRP3 inflammasome, causing secretion of IL-1*β* and IL-18 [73]. The authors postulated that NLRP3 may be a sensor for drusen-induced inflammasomes, as NLRP3 has been shown previously to act as a receptor for “danger” signals such as amyloid-like structures. Because laser-induced CNV was considerably greater in NLRP3 knockout mice, but not IL-1R knockout mice, NLRP3 and IL-18 may have a protective role in the progression of AMD [73]. Further, CEP (carboxyethylpyrrole), a biomarker of AMD, was thought to prime the inflammasome. Interestingly, while C1q, another complement component known to contribute to the inflammation and the pathophysiology of AMD [61], can also act as a danger signal that is, sensed by the NLRP3 inflammasome [75], C1q knockout mice develop CNV of similar size to control mice [61]. In addition, a recent study reported that lysosomal destabilization can activate the NLRP3 inflammasome in RPE cells [76]. 

 Regulation of the NLRP3 inflammasome is poorly understood but probably involves the integration of signals from a number of stimuli, such as cellular damage and stress. It is now appreciated that inflammasome-dependent biological effects may be mediated not only by IL-1*β* and IL-18, but also by the multifaceted activities of caspase-1. Therefore, it is important to determine the mechanisms by which inflammasomes in RPE cells directly or indirectly modulate IL-1*β* activity that may lead to AMD. In chronically stressed states, where autophagy is increased, there may be secondary effects of protecting against inflammasome activation [77, 78]. Further understanding in context of drusen and RPE behavior may provide pathways to interrogate to maintain RPE function and health and attenuate inflammatory activation. Future studies to better understand how inflammasomes may be activated in AMD, and the molecular mechanisms involved in the assembly of the inflammasome signaling platform, may therefore lead to the development of novel therapeutic approaches for AMD.

## 4. Para-Inflammation in AMD 

Inflammation, both acute and chronic, functions to control danger signals or to respond to pathogens to safeguard a host and maintain tissue health. Disturbances of homeostasis (e.g., infection, tissue injury, foreign bodies, but may also include stresses from aging) trigger inflammatory responses, the purpose of which are to remove or sequester the source of the disturbance and to allow the host to adapt to the abnormal conditions and return to a state of homeostasis. However, the spectrum of inflammation is broad. When appropriate, inflammation can be both adaptive and protective. Conversely, the immune response also has significant pathological potential and can promote tissue damage and facilitate disease progression.

 Medzhitov first introduced the idea of para-inflammation as a tissue adaptive response to noxious stress or malfunction that has characteristics intermediate between basal and inflammatory states [10]. Briefly, in the basal state, tissue-resident macrophages (principally retinal microglia and retinal perivascular macrophages or choroidal macrophages) may play a role to promote an adaptive change with short-term benefits, promoting tissue homeostasis. However, if the abnormal conditions are sustained, or if the tissue receives a “danger signal,” this can result in immune cell infiltration, which in turn can become maladaptive. Para-inflammation has characteristics that are intermediate between basal and inflammatory states. The purpose of normal para-inflammation is presumably to maintain tissues homeostasis and to restore tissue function. Nonetheless, if a tissue is exposed to prolonged stress or malfunction, para-inflammation can become chronic and promote disease progression. Dysregulated para-inflammation has been proposed to play an important role in the progression of diabetes, atherosclerosis, and obesity.

 Similarly, dysregulated para-inflammation, which is especially relevant in aging tissues dependent on nonproliferative cells and characterized by very high metabolism and other oxidative stress (e.g., the macula), has also been postulated to contribute to the development of AMD [11]. In the aging retina, oxidized lipoproteins and free radicals are major causes of tissue stress and serve as local triggers for retinal para-inflammation. Para-inflammatory responses in the neuroretina may be reflected in microglial activation and subretinal migration, and (potentially) breakdown of blood-retinal barrier. At the retinal/choroidal interface, para-inflammation manifests as complement activation in Bruch's membrane and RPE cells, and accumulation of microglia (and myeloid cells that have recently immigrated) in the subretinal space. In the choroid, para-inflammation may be characterized by increased thickness of choroid, increased macrophages, morphological abnormalities in choroidal melanocytes, mast cell activation and fibrosis.

 Recent evidence, derived from the cybrid models of mitochondrial haplotypes into a mitochondrial DNA-null RPE cell line (ARPE19), showed that mitochondrial dysfunction may promote the progression and AMD [79]. The observed distinct polarization of energy cellular energy source and production suggest an approach with promise in further interrogating the influence on immune responses, including para-inflammation. The notion is that switching energy sources, which may be dependent on haplotype, influences the signaling pathways and thus phenotype of any subsequent immune activation of the cell. Further studies may increase our understanding of potential switch of energy sourcing, and the influence on immune activation of RPE that in turn will direct immune responses in cells (i.e., macrophages and choroidal mast cells) to deliver a trigger for progression of disease.

## 5. Adaptive Immunity in AMD

 The role of adaptive immunity in AMD has received increasing attention. Whether adaptive immune responses relay pathogenic or regulatory functions, or are simply bystander effects, remains elusive. In support, there have been numerous reports suggesting involvement due to finding of autoantibodies in AMD patients, not least with the detection of anti-retinal autoantibodies [80, 81]. Whether they have a role as potential pathogenic mediators, or occur as bystanders, it remains to be determined if autoantibodies can act as a prognosticator or biomarker in AMD patients [82]. The search has been driven further with utilization of serum antigen arrays and 2-D gel electrophoresis. Specific targets such as RPB-3, aldolase C and pyruvate kinase IgG have been derived, and altered IgG/IgM ratios of anti-phophsatidylserine are associated with patients with AMD [83, 84]. Autoantibodies have been observed even when investigating responses to complement regulators, such as CFH [85]. The latter finding is enticing in that autoantibodies to CFH were unexpectedly lower in AMD patients, inferring a protective effect. Nevertheless, together there is increasing evidence of the presence of autoantibodies in AMD. The spectrum suggests secondary effects, and indeed also infers the potential of adaptive immune engagement. Consequently further searches for autantibodies, albeit possibly in only a small subset of patients, may be justified to determine whether there is a prevalent autoantibody signature.

 More compelling data arises from mouse work. The data from Hollyfield et al. [43, 86] demonstrated that carboxyethylpyrrole (CEP) is present in AMD eye tissue, and mice immunized with this adducted oxidated product generated antibodies and exhibited pathology with some similarities with human AMD. Moreover, in experiments in RAG-deficient animals which lack B and T cells, no anti-CEP antibody was detected. Given the cell infiltrate noted around lesions, both T cell engagement and complement fixation were thought to contribute in this model to the loss of RPE and photoreceptors, and thus progression of AMD.

 Most recently, novel observations of cytokine and T cell signatures from AMD patients have been published. First, an intriguing increase in IL-22 and IL-17 levels in serum from AMD patients was shown, supported by the further finding that C5a stimulated IL-22 and IL-17 from T cells [87]. Second, studies of twins and siblings found that the *IL-17RC* promoter is hypomethylated in AMD patients [88], further suggesting the involvement of adaptive immunity and TH17 cells, as well as potential effect on macrophages. Consequently, a testable hypothesis is that autoantibodies are present early in subsets of AMD patients, and are pathogenic. The notion that autoantibodies may create further complement-mediated damage, or activate myeloid cells to switch from protective para-inflammatory to pathogenic responses, may also be tested. Generation of autoantibodies (i.e., engagement of adaptive immune responses that are pathogenic) may tip the balance from para-inflammatory control, and create an environment that induces further loss of cells, angiogenesis, and an unremitting walk to late stage AMD.

## 6. Summary of BIMR Conference Discussions

 Recent data suggest that dysregulation of immune response could contribute to the pathogenesis of AMD. However, there are a number of questions that remain unanswered. First, if para-inflammation is involved in the pathogenesis of AMD, when and how does a dysregulation of the immune response change from a protective role to a harmful process? Second, although genetic studies point to a role for the complement system and innate immunity in AMD, what role if any does adaptive immunity play in the disease? The group discussed a number of experimental approaches that could help address these questions. 

### 6.1. Human Tissue Studies

Existing tissue banks may be used to interrogate immune response in AMD, with a particular focus on early events. Diseased and fellow eye tissue might first be graded using an established system [89], and then comprehensively characterized in terms of inflammatory cell contents and patterns, presence of complement and autoantibodies, and gene and protein expression profiles. It may be especially useful to compare tissue from different areas of an individual retina (e.g., in the fovea, adjacent to drusen, and “normal” tissue away from drusen). 

### 6.2. Retrospective Clinical Studies

Existing medical records from large databases, such as those available from Medicare in the USA or anti-TNF-*α* treatment registries in the UK, could be mined to gain insight into selected questions. For example, do patients on immunosuppressive therapies for rheumatologic diseases have a lower prevalence of AMD?

### 6.3. Prospective Clinical Trials

There are a number of randomized clinical trials examining immunomodulation as a therapy for retinal diseases including AMD. Results from these trials will help guide our knowledge about the role of the immune system in the disease. Examples of such informative trials include prior trials studying treatment with immunomodulators in other diseases, and trials in AMD using immunomodulators such as those that target C5 and other complement components, mTOR, or TNF-*α*.

### 6.4. Biomarkers

Identification of direct and/or surrogate biomarkers that are predictive or prognostic for disease susceptibility, disease progression, or treatment response will be beneficial to the study of AMD. Serum, plasma, PBLs, platelets, and aqueous humor could be obtained from patients enrolled in natural history studies, and at the same time, patients encouraged to consent to eventually donate eyes. Samples could be used to assess complement components, cytokines, carboxyethylpyrrole (CEP), and autoantibodies. Collectively, such studies may yield clinical/pathological correlations and genotype/phenotype relationships in individual patients.

### 6.5. Imaging Modalities

The development of new imaging modalities can detect the trafficking and function of immune cells in the retina and choroid (which may be transient). These tools, if designed to provide quantitative analyses of immune system functions such as the presence in the human eye of ongoing complement activation or specific cellular infiltration could then be applied to both direct imaging studies, particularly immunomodulatory treatments and bioenergetic evaluations, and to studies using transfer of ex vivo labeled cells.

### 6.6. Animal Models

Although there is no perfect animal model for AMD, preclinical models that reproduce specific aspects of early AMD (e.g., drusen, low grade chronic inflammation, and GA) need to be developed to ask specific questions about the role of inflammation, and to probe specific disease mechanisms. Although eyes in mice do not have macular structures and do have a distinct RPE morphology, an example of a model for para-inflammation may include deliberately inducing low grade inflammation in* ob/ob* or senescent mice, followed by addition of a systemic insult (e.g., light toxicity) to reproduce the hypothesized dysregulation of para-inflammation.

### 6.7. In Vitro Cell Biology Studies

Finally, there is interest in studies designed question whether changes in aging RPE and photoreceptors make them more susceptible to damage by dysregulated para-inflammation in AMD. For example, in vitro cultures of photoreceptors could be used to evaluate early changes in rods in AMD, or resistance to injury by cones in AMD. Cultures of RPE could be used to examine metabolic dysfunctions (e.g., mitochondrial dysfunction, haplotypes), and the responses of retinal and choroidal cells to cytokines released by such metabolic change. Of particular interest are epigenetic changes in such cells that may promote or protect cells in aging and AMD.

## 7. Conclusions

**Figure 2:**
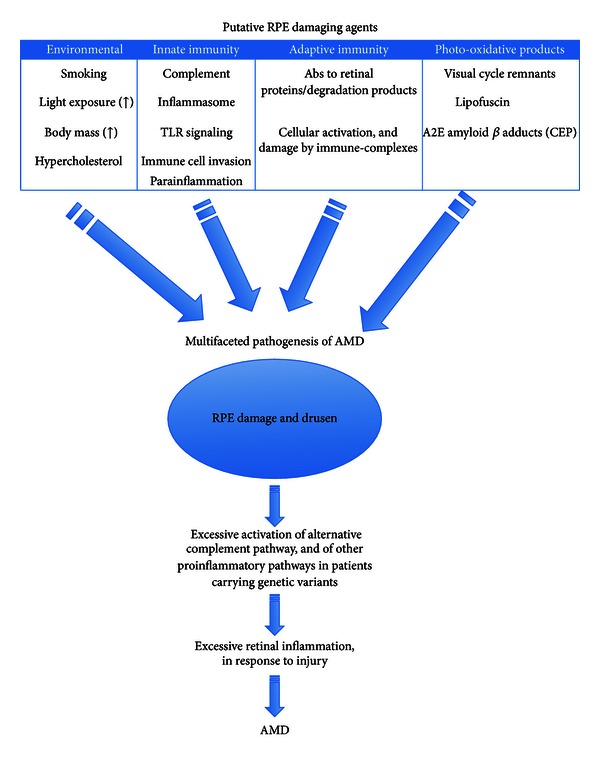
An integrated model of immune regulation of AMD.

 The prevalence of AMD will continue to increase as the population ages. Although we do not know the exact etiology of the disease, recent genetic studies have implicated the complement system in disease pathogenesis and severity. Other studies further support the hypothesis that the immune system is involved in the disease, in concert with or in addition to other factors such as environmental conditions and products of photooxidation (Figure 2). Importantly, understanding how immune responses initiate or exacerbate AMD will allow us to identify novel therapeutic approaches to the disease.
